# Impacts of ivermectin mass drug administration for onchocerciasis on mosquito populations of Ogun state, Nigeria

**DOI:** 10.1186/s13071-021-04716-3

**Published:** 2021-04-20

**Authors:** Olaitan Olamide Omitola, Cynthia Uchechukwu Umunnakwe, Adedotun Ayodeji Bayegun, Samuel Akinjide Anifowose, Hammed Oladeji Mogaji, Akinola Stephen Oluwole, Simon Nnayere Odoemene, Taiwo Sam Awolola, Adebola Adedoyin Osipitan, Sammy Olufemi Sam-Wobo, Uwem Friday Ekpo

**Affiliations:** 1 Department of Pure and Applied Zoology, Federal University of Agriculture, Abeokuta, Ogun Nigeria; 2 Department of Animal and Environmental Biology, Federal University Oye Ekiti, Ekiti, Nigeria; 3 COUNTDOWN Consortium, Sightsavers Nigeria Country Office, Kaduna, Nigeria; 4 Department of Biological Sciences, Adeleke University, Ede, Osun Nigeria; 5 Molecular Entomology and Vector Control Research Laboratory, Public Health Division, Nigeria Institute of Medical Research, Lagos, Nigeria; 6 Department of Crop Protection, Federal University of Agriculture, Abeokuta, Ogun Nigeria

**Keywords:** Ivermectin, Endectocide, Mosquito, Vector control, Nigeria

## Abstract

**Background:**

The impact of single-dose mass drug administration (MDA) of ivermectin for onchocerciasis on mosquito populations was investigated in Ogun State, Nigeria.

**Methods:**

Indoor and outdoor collection of mosquitoes was carried out in two intervention (IC) and two control communities (CC) at three different periods: pre-MDA (baseline), 2–3 days after MDA and 13–14 days after MDA. The density and parity rate of female *Anopheles* and *Culex* mosquitoes were determined and compared. Environmental and climatic data of study locations were obtained to perform generalized linear model analysis.

**Results:**

A total of 1399 female mosquitoes were collected, including 1227 *Anopheles* and 172 *Culex* mosquitoes. There was a similar magnitude of reduction in the indoor density of *Anopheles* by 29% in the IC and CC 2–3 days post-MDA but the reduction in indoor parity rate was significantly higher (*p* = 0.021) in the IC, reducing by more than 50%. In the IC, observation of a significant reduction at 2–3 days post-MDA was consistent for both the indoor density (1.43 to 1.02) and indoor parity rate (95.35% to 44.26%) of *Anopheles* mosquitoes. The indoor parity rate of *Anopheles* remained significantly reduced (75.86%) 13–14 post-MDA. On the other hand, the indoor density of *Culex* increased from 0.07 to 0.10 at 2–3 days post-MDA while the indoor parity rate of *Culex* did not change. The outdoor density of *Anopheles* in the IC increased (*p* = 0.394) from 0.58 to 0.90 at 2–3 days post-MDA; a similar observation was consistent for the outdoor density (2.83 to 3.90) and outdoor parity rate (70.59% to 97.44%) of *Culex*, while the outdoor parity rate of *Anopheles* reduced from 85.71 to 66.67% at 2–3 days post-MDA. A generalized linear model showed that ivermectin MDA significantly caused a reduction in both the indoor density (*p* < 0.001) and indoor parity rate (*p* = 0.003) of *Anopheles* in the IC.

**Conclusion:**

Ivermectin MDA resulted in the reduction of both the survival and density of *Anopheles* mosquitoes. This has strong implications for malaria transmission, which depends strongly on vector survival.

**Graphic abstract:**

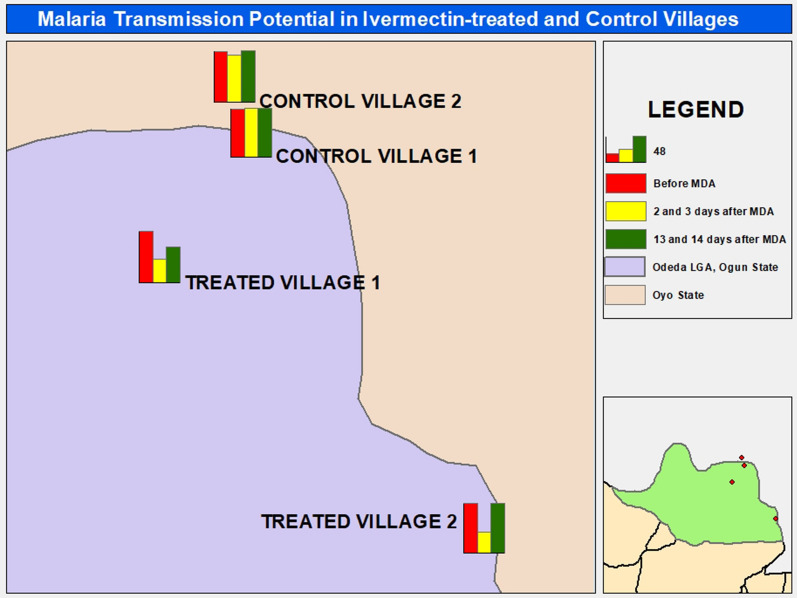

## Introduction

Mosquitoes alone account for 17% of the estimated global burden of infectious diseases, and every year, malaria which is transmitted by the *Anopheles* mosquito, causes the deaths of more than 400,000 people and incapacitates another 200 million for days [1]. In Nigeria, malaria accounts for about 11% maternal mortality, 25% infant mortality, 30% under-5 mortality, 60% outpatient visits and 30% hospital admissions [2]. To avert the deaths and morbidity caused by malaria and other mosquito-borne diseases, vector control has been adopted as the mainstay of control strategies. Current methods involve the deployment of insecticides to eliminate biting or resting mosquitoes by mounting long-lasting insecticidal nets (LLINs) where the mosquito may land when trying to reach the person sleeping under the net and spraying walls inside the house where blood-fed mosquitoes will often rest [3]. However, mosquitoes are still able to escape these strategies because they bite when people are not under LLINs or because they rest or feed outside the sprayed house.

Interest in the development of interventions that are capable of targeting mosquitoes that can escape the current strategies for vector control is emerging. The adoption of ivermectin, used in the treatment of filarial worm infections, has been proposed to address limitations of the current malaria control tools such as residual transmission [4]. Residual transmission occurs when malaria continues to be transmitted, despite the effective implementation of available vector control tools, as a result of mosquitoes adapted to biting outside the time of the day when LLINs and indoor residual spraying can protect people. Ivermectin is a systemic endectocide, which, when administered to humans or animals, is also toxic to mosquitoes that feed on the treated vertebrate host. This provides an opportunity to eliminate mosquitoes that may escape or survive existing vector control interventions since all female mosquitoes must obtain blood meals for the development of their eggs [3].

Studies have reported the ability of ivermectin to reduce the life span and vectorial capacity of *Anopheles* sp. that feed on treated humans [4, 5]. Although it is shown that *Anopheles* mosquitoes captured from villages after ivermectin MDA have reduced lifespans when monitored in the laboratory, this does not adequately represent the mass effect on mosquito populations under real-life settings. There is a need to evaluate how the effect of ivermectin on mosquito lifespan impacts the density or abundance of the mosquito population in a locality.

Besides, generating local evidence of the mosquitocidal potentials of ivermectin mass drug administration will assist in effective targeting and optimized delivery of the drug in the context of vector control in endemic areas. Findings suggest the extent of the mosquitocidal effect of ivermectin may vary within the anopheline group [4]. Hence, it is important to understand how the local vector population of a geographical area, which comprises a unique diversity of primary and secondary vector species, is affected during ivermectin mass drug administration. In this study, we investigated the impacts of the annual round of ivermectin MDA, used for the control of onchocerciasis and lymphatic filariasis (LF), on local mosquito vector populations in the communities of Odeda local government area (LGA) in Ogun State, Nigeria.

## Methods

### Study area

**Fig. 1:**
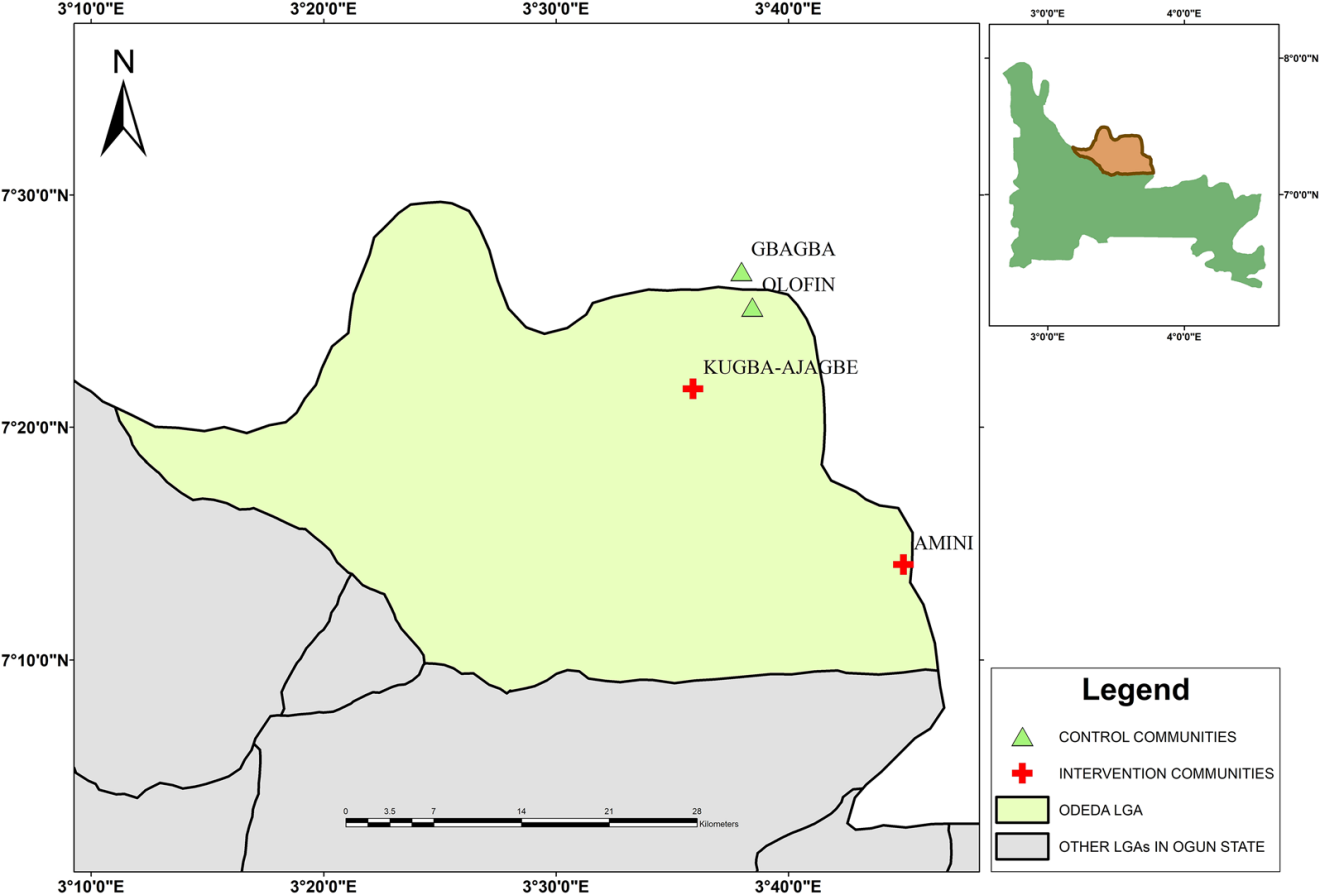
Map of the study area showing the Ogun State communities where the study was conducted

This study was carried out in Odeda Local Government Area (LGA) of Ogun State, Nigeria, between August and September 2018. Odeda LGA is endemic for onchocerciasis and LF, where community-directed treatment with ivermectin (CDTI) is being implemented annually through mass drug administration (MDA) with single-dose administration of 150–200 μg/kg ivermectin (Mectizan®, Merck & Co Inc.). Treatment coverage was between 66 and 80% in the LGA between 2013 and 2017 according to the ivermectin therapeutic coverage records of the Ogun State Ministry of Health (SMoH). Four communities—Kugba-Ajagbe (7.36102° N, 3.59830° E) and Amini (7.23509° N, 3.74935° E) as intervention communities and Olofin (7.41974° N, 3.64086° E) and Gbagba (7.44530° N, 3.63307° E) as control communities—were selected for the study. The intervention communities have been receiving ivermectin MDA rounds consistently from 2013 to 2017, whereas the control communities have no records of ivermectin treatment (Fig. 1). The four communities are a part of the sparsely dispersed settlements in the border region between Ogun and Oyo States of Nigeria. Each community is located about 3 km from the nearest villages in the surrounding area.

### Study design

**Fig. 2:**
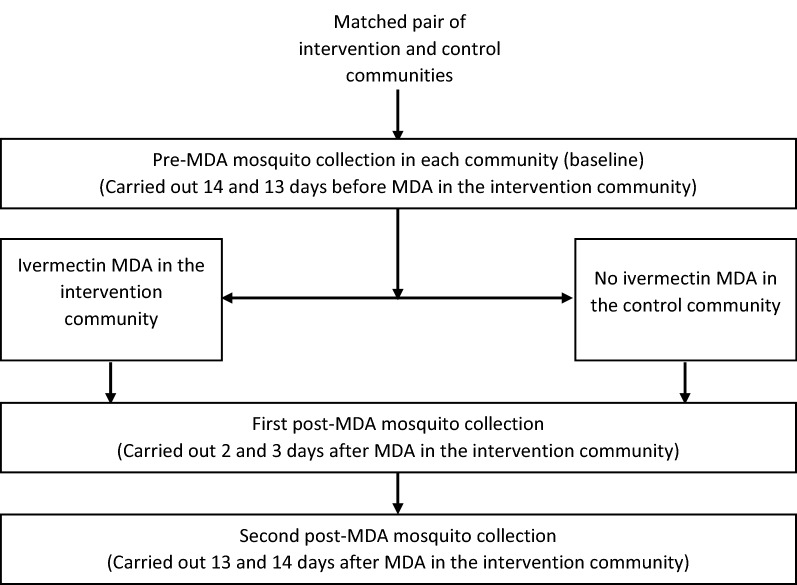
The study design for mosquito collection

The cross-sectional study involved the collection of mosquito samples from each community at three different sampling intervals in Fig. 2.

In each community, 15 houses were enumerated for the collection of indoor-resting mosquitoes using systematic selection, beginning with the village head’s resident as the reference house. Where consent could not be obtained, the next consenting household was selected. Three outdoor locations, where inhabitants commonly gather in the evenings, were also selected in each community for outdoor mosquito collection [6]. Sampling intervals were spaced to allow sufficient time for recoil in the mosquito populations and minimize the effect of the mosquito collection on the population density. MDA with ivermectin was carried out by the Ogun State neglected tropical diseases (NTD) control programme on 3 and 10 September 2018 in Kugba-Ajagbe and Amini, respectively. The surrounding villages of both the intervention and control communities received ivermectin MDA during the study.

### Collection, identification and dissection of mosquitoes

Mosquitoes were collected indoors between 05.00 and 09.00 h in the morning by adapting the pyrethrum spray catch (PSC) method previously described [7], with modifications including the use of the commercial aerosol Raid® (containing 0.250% allethrin, 0.150% tetramethrin, 0.015% deltamethrin and 99.585% inert ingredients). This was sprayed in each room by two collectors, who returned after 10 min to retrieve the knocked-down mosquitoes. CDC Miniature Light Traps (Model 512; John W. Hock Co., Gainesville, FL, USA) were used as a convenient and cheap alternative to human-landing catch (HLC) for outdoor collection of mosquitoes because of the ethical concerns and labour involved in HLC [8, 9]. During each mosquito collection visit to a community, three light traps were set up in three outdoor locations at about 18.00 h at dusk and retrieved at about 06.00 h at dawn. A bottle, containing a mixture of yeast and sugar solution, was hung near each trap as CO_2_ bait. Mosquitoes were collected from the rooms and traps into labelled Petri dishes and the mosquito samples were transported to the laboratory at the Department of Pure and Applied Zoology. The female mosquitoes were sorted and identified to genus level using morphological keys [10]. All the female *Anopheles* and *Culex* mosquitoes collected were dissected for age grouping (parity status) based on the ovarian tracheoles [6, 11].

### Entomological parameters

Mosquito population characteristics were measured in terms of density (abundance) and parity rate (age structure). The indoor density of mosquitoes was calculated using the formula below:

$${\text{Density}} = \frac{{{\text{Number}}\;{\text{of}}\;{\text{female}}\;{\text{mosquitoes}}\;{\text{collected}}}}{{{\text{Number}}\;{\text{of}}\;{\text{rooms}} \times {\text{Number}}\;{\text{of}}\;{\text{nights}}}}.$$

The outdoor density of mosquito was calculated using the formula below:

$${\text{Density}} = \frac{{{\text{Number}}\;{\text{of}}\;{\text{female}}\;{\text{mosquitoes}}\;{\text{collected}}}}{{{\text{Number}}\;{\text{of}}\;{\text{traps }} \times {\text{Number}}\;{\text{of}}\;{\text{nights}}}}.$$

Parity rate was also calculated as shown below:

$${\text{Parity rate (\%)}} = \frac{{{\text{Number of parous female mosquitoes}}}}{{{\text{Total number of female mosquitoes}}}} \times 100.$$

### Determination of ivermectin MDA coverage

MDA coverage was determined 3–4 weeks after MDA in the intervention communities using the coverage survey builder (CSB) protocol and a questionnaire developed by the WHO [12]. The number of households surveyed was calculated with the CSB, estimated at 15 households per community. The questionnaire was administered, and respondents were shown a sample of the drug package to facilitate recall during the interviews. Interviews were conducted by translating the questionnaire into the local language. Every member of the selected household was interviewed, regardless of their age or eligibility for MDA. Where a household member was absent or too young to respond personally, the household head or an available adult member of the household responded on their behalf.

### Remote-sensing climatic and environmental data

Geographic coordinates of the communities were obtained with a portable GPS receiver (eTrex®10, Garmin™ International, Olathe, KS, USA). Rainfall and vegetation index data were collected as possible confounders on mosquito populations during the study. Data for precipitation and the normalized difference vegetation index (NDVI) were sourced from two open-access satellite imagery databases: EarthExplorer [13] and Precipitation Estimation from Remotely Sensed Information using Artificial Neural Networks (PERSIANN), respectively [14]. For each mosquito collection visit, available data for 14 days up to the collection date were retrieved since mosquito development lasts for about 5–14 days.

### Data analyses

Secondary data from remote sensing resources were processed using the HDF-EOS to GeoTIFF (HEG) Conversion Tool (Raytheon Co., Riverdale, MD, USA) and ArcGIS 10.3 (ESRI Incorp., Redlands, CA, USA) was used to extract raster values for the relevant climatic and environmental variables. These data, together with primary data from mosquito collection and coverage survey, were inputted into a Microsoft Office Excel spreadsheet (Microsoft Corp., Redmond, WA, USA) and imported into IBM SPSS Statistics (IBM Corp., Somers, NY) for statistical analyses. Data were subjected to descriptive statistics to present frequency, total, mean and percentage tables. Mann-Whitney *U* test and chi-square goodness-of-fit test were used to compare mosquito abundance between sampling intervals within a community and between study areas. Pearson chi-square test was used to compare mosquito parity rates between the sampling intervals and to compare MDA coverage between communities. Ivermectin exposure was graded into (1) early post-MDA, (2) late Post-MDA and (3) pre-MDA zero exposure, based on the relative degree to which MDA was expected to impact the mosquito populations at the different sampling intervals. A generalized linear model was applied to determine the effects of MDA alone as well as the effects of MDA alongside other factors on the density and parity rate of the mosquitoes in the intervention communities. In all the instances of statistical analyses, a *p*-value < 0.05 was used to determine statistical significance.

## Results

### Composition and distribution of the mosquito samples collected

A total of 1399 mosquitoes were collected across the study communities: 1227 (87.7%) were *Anopheles* sp. and 172 (12.3%) were *Culex* sp. Of the 1227 *Anopheles* sp. collected, 975 (79.4%) were from the control communities while 109 (63.4%) of the 172 *Culex* sp. were collected from the intervention communities. Among the *Anopheles* sp. collected, a total of 1109 (90.4%) and 118 (9.6%) were indoor and outdoor resting mosquitoes, respectively. Similarly, among the *Culex* sp. a total of 135 (78.5%) and 37 (21.55) were outdoor and indoor resting mosquitoes, respectively (Table 1).

**Table 1:** Descriptive statistics of the mosquito samples collected

	Intervention		Control		Total	
	No. of mosquitoes	Parous mosquitoes	No. of mosquitoes	Parous mosquitoes	No. of mosquitoes	Parous mosquitoes
	*N* (%)	*N* (parity)	*N* (%)	*N* (parity)	*N* (%)	*N* (parity)
*Anopheles* sp.						
Indoor	234 (92.9)	175 (74.79)	875 (89.7)	792 (90.51)	1109 (90.4)	967 (87.20)
Outdoor	18 (7.1)	12 (66.67)	100 (10.3)	68 (68.00)	118 (9.6)	80 (67.80)
Total	252	187	975	860	1227	1047
*Culex* sp.						
Indoor	25 (22.9)	24 (96.00)	12 (19.0)	11 (91.67)	37 (21.5)	35 (94.60)
Outdoor	84 (77.1)	72 (85.71)	51 (81.0)	41 (80.39)	135 (78.5)	113 (83.70)
Total	109	96	63	52	172	148
Overall total	361	283	1038	912	1399	1195 (85.42)

### Comparison of mosquito density between sampling intervals

**Fig. 3:**
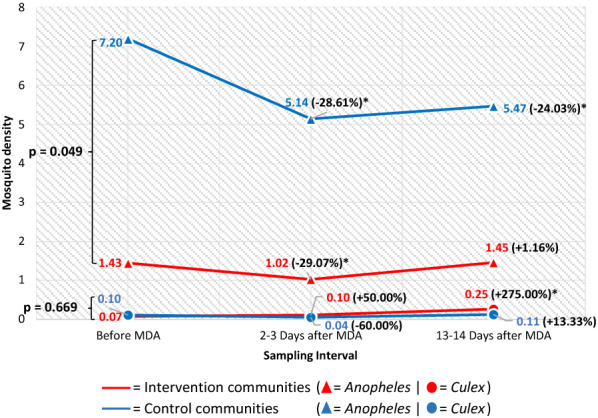
Indoor density of *Anopheles* and *Culex* in the intervention and control communities across sampling intervals. *Statistically significantly lower or higher density than before MDA

The density of mosquitoes collected indoors at the sampling intervals in the intervention and control communities is shown in Fig. 3. Before MDA, the indoor density of *Anopheles* sp. was significantly higher (*p* = 0.043) in the control communities (7.20) than in the intervention communities (1.43). At 2–3 days post-MDA, the density reduced in both the intervention and control communities by a factor of 29.07% and 28.61%, without a significant difference between both reductions (*p* = 0.561). In the intervention communities, the indoor density of *Anopheles* sp. reduced significantly (1.02, *p* = 0.039) 2–3 days post-MDA but increased 13–14 days post-MDA (1.45, *p* = 0.939) compared to pre-MDA. In the control communities, this was 5.14 and 5.47 at 2–3 days and 13–14 days post-MDA, both significantly (*p* < 0.001) lower than before MDA. The difference between the indoor density of *Culex* sp. in the control and intervention communities before MDA was not statistically significant (0.10 *vs* 0.07, *p* = 0.752). In the intervention communities, this increased 2–3 days (0.10, *p* = 0.527) and 13–14 days post-MDA (0.25, *p* = 0.012) compared to pre-MDA. In the control communities, this reduced 2–3 days post-MDA (0.04, *p* = 0.257) and increased 13–14 days post-MDA (0.11, *p* = 0.849) compared to pre-MDA.

**Fig. 4:**
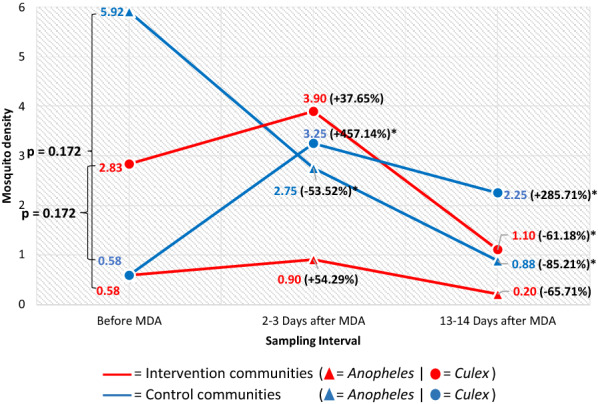
Outdoor density of *Anopheles* and *Culex* in the intervention and control communities across sampling intervals. *Statistically significantly lower or higher density than before MDA

In Fig. 4, the densities of mosquitoes collected outdoors at the sampling intervals are shown. Before the MDA, the difference in the outdoor density of *Anopheles* sp. between the control and intervention communities was not statistically significant (5.92 *vs* 0.58, *p* = 0.465). In the intervention communities, this increased 2–3 days post-MDA (0.90, *p* = 0.394) and reduced 13–14 days post-MDA (0.20, *p* = 0.160) compared to before MDA. In the control communities, this reduced significantly 2–3 days (2.75, *p* = 0.001) and 13–14 days post-MDA (0.88, *p* < 0.001) compared to pre-MDA. The difference between the outdoor density of *Culex* sp. in the control and intervention communities before MDA was also not statistically significant (0.58 *vs* 2.83, *p* = 0.053). In the intervention communities, this increased 2–3 days post-MDA (3.90, *p* = 0.173) and reduced significantly 13–14 days post-MDA (1.10, *p* = 0.005) compared to pre-MDA. In the control communities, the density at 2–3 days (3.25) and 13–14 days post-MDA (2.25), was significantly higher than pre-MDA (*p* < 0.001 and *p* = 0.001 respectively).

### Comparison of mosquito parity rate between sampling intervals

**Fig. 5:**
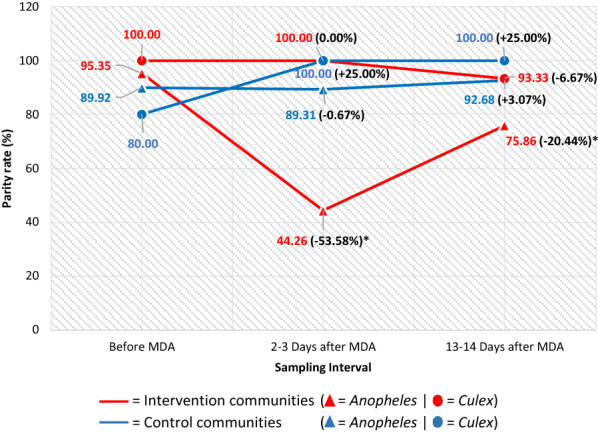
Parity rate of *Anopheles* and *Culex* collected indoors in the intervention and control communities. *Statistically significantly lower or higher parity rate than before MDA

Figure 5 illustrates the parity rate of the mosquitoes collected indoors. The percentage reduction in the indoor parity rate of *Anopheles* sp. 2–3 days post-MDA was significantly higher (*p* = 0.021) in the intervention (53.58%) than in the control communities (0.67%). In the intervention communities, the indoor parity rate of *Anopheles* sp. reduced significantly 2–3 days (44.26%, *p* < 0.001) and 13–14 days post-MDA (75.86%, *p* = 0.001) compared to 95.35% before MDA. In the control communities, this reduced 2–3 days post-MDA (89.31%, *p* = 0.910) and increased 13–14 days post-MDA (92.68%, *p* = 0.303) compared to 89.92% before MDA. Similarly, the indoor parity rate of *Culex* sp. in the intervention communities was 100.00% pre-MDA and did not change significantly 2–3 days (100.00%) and 13–14 days post-MDA (93.33%, *p* = 0.789). In the control communities, this increased 2–3 days (100.00%, *p* = 0.714) and 13–14 days post-MDA (100.00%, *p* = 0.500) compared to 80.00% before MDA.

**Fig. 6:**
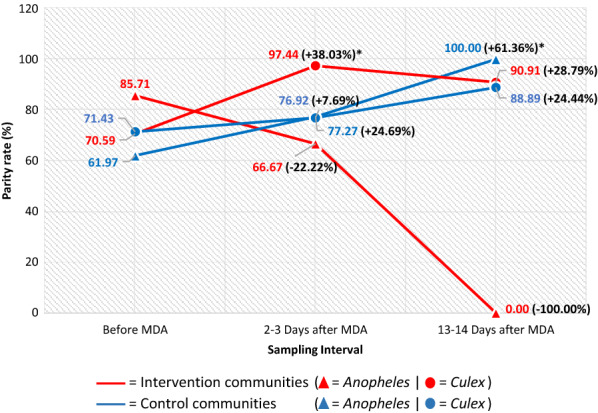
Parity rate of *Anopheles* and *Culex* collected outdoors in the intervention and control communities. *Statistically significantly lower or higher parity rate than before MDA

In Fig. 6, the parity rate of *Anopheles* sp. collected outdoors in the intervention communities reduced 2–3 days (66.67%, *p* = 0.392) and 13–14 days post-MDA (0.00%, *p* = 0.083) from 85.71% before MDA. In the control communities, this increased 2–3 days (77.27%, *p* = 0.288) and 13–14 days post-MDA (100.00%, *p* = 0.044) from 61.97% before MDA. The outdoor parity rate of *Culex* sp. in the intervention communities was higher at 2–3 days (97.44%, *p* = 0.004) and 13–14 days post-MDA (90.91%, *p* = 0.170) compared to 70.59% before MDA. In the control communities, this increased 2–3 days (76.92%, *p* = 0.556) and 13–14 days post-MDA (88.89%, *p* = 0.307) compared to 71.43% pre-MDA.

### MDA coverage and changes in mosquito populations in the intervention communities

The coverage evaluation survey showed that MDA coverage was significantly (*p* = 0.024) higher in Amini than Kugba-Ajagbe (Table 2). MDA coverage in the two intervention communities, as well as changes in the density and parity rate of *Anopheles* and *Culex* populations, are also shown in Table 2.

**Table 2:** Ivermectin MDA coverage and mosquito population changes in the intervention communities

		*Kugba-Ajagbe*	*Amini*	*p*-value
N^o^ of eligible persons surveyed				
Female		32	23	
Male		29	18	
Total		61	41	
N^o^. of persons treated (%)				
Female		17 (68.0)	14 (51.9)	
Male		8 (32.0)	13 (48.1)	
Total coverage		25 (41.0)	27 (65.9)	0.024*
*Anopheles* sp.				
Indoor density	14–13 days before MDA	1.97	0.90	
	2–3 days post-MDA	1.70 (*p* = 0.446)	0.33 (*p* = 0.005)^†^	
	13–14 days post-MDA	1.93 (*p* = 0.926)	0.97 (*p* = 0.789)	
Outdoor density	14–13 days before MDA	1.00	0.17	
	2–3 days post-MDA	0.83 (*p* = 0.763)	1.00 (0.068)	
	13–14 days post-MDA	0.33 (*p* = 0.157)	0.00	
Indoor parity rate	14–13 days before MDA	96.61	92.59	
	2–3 days post-MDA	45.10 (*p* < 0.001)^†^	40.00 (*p* = 0.002)^†^	
	13–14 days post-MDA	67.24 (*p* < 0.001)^†^	93.10 (*p* = 0.667)	
Outdoor parity rate	14–13 days before MDA	83.33	100.00	
	2–3 days post-MDA	80.00 (*p* = 0.727)	50.00 (*p* = 0.600)	
	13–14 days post-MDA	0.00 (*p* = 0.107)	–	
*Culex* sp.				
Indoor density	14–13 days before MDA	0.13	0.00	
	2–3 days post-MDA	0.07 (*p* = 0.414)	0.13	
	13–14 days post-MDA	0.23 (*p* = 0.366)	0.27	
Outdoor density	14–13 days before MDA	1.50	4.17	
	2–3 days post-MDA	5.33 (*p* < 0.001)^†^	1.75 (*p* = 0.036)^†^	
	13–14 days post-MDA	1.50 (*p* = 1.000)	0.50 (*p* = 0.001)^†^	
Indoor parity rate	14–13 days before MDA	100.00	–	
	2–3 days post-MDA	100.00	100.00	
	13–14 days post-MDA	100.00	87.50	
Outdoor parity rate	14–13 days before MDA	88.89	64.00	
	2–3 days post-MDA	96.88 (*p* = 0.395)	100.00 (*p* = 0.073)	
	13–14 days post-MDA	88.89 (*p* = 0.765)	100.00 (*p* = 0.436)	

*Difference between total MDA coverage is statistically significant

^†^Density or parity rate is statistically significantly higher or lower than before MDA

### Data for rainfall and vegetation index

In all the communities, average rainfall progressively increased from the period before MDA to the period after MDA (Table 3). The normalized difference vegetation index (NDVI) also increased progressively in Kugba-Ajagbe and Gbagba. However, changes in the NDVI in Amini and Olofin were contrasting and not similar.

**Table 3:** Data for rainfall and vegetation index across the sampling intervals

	14–13 days before MDA	2–3 days post-MDA	13–14 days post-MDA
Rainfall (mm)			
Kugba-Ajagbe	0.2760	2.2273 (0.028*)	4.5480 (0.018*)
Amini	0.8827	3.9467 (0.040*)	4.7820 (0.071)
Olofin	0.2539	1.4413 (0.093)	5.3407 (0.012*)
Gbagba	0.7887	4.7653 (0.033*)	5.2660 (0.018*)
Vegetation index			
Kugba-Ajagbe	0.2535	0.5298 (< 0.001*)	0.5862 (< 0.001*)
Amini	0.1872	0.4779 (< 0.001*)	0.4156 (< 0.001*)
Olofin	0.2269	0.1118 (< 0.001*)	0.5318 (< 0.001*)
Gbagba	0.2535	0.5756 (< 0.001*)	0.6360 (< 0.001*)

*Rainfall or vegetation index is statistically significantly lower or higher than before MDA

### Effects of ivermectin MDA, rainfall and vegetation index on the mosquito density and parity rate in the intervention communities

In Table 4, the generalized linear model showed that ivermectin MDA alone had a significant effect on the parity rate of indoor (*p* < 0.001) and outdoor (*p* < 0.001) *Anopheles* populations in the intervention communities but no significant effects on density. Ivermectin MDA alone also showed a significant effect on the density of indoor (*p* = 0.005) and outdoor (*p* = 0.041) *Culex* populations in the intervention communities but no significant effects on parity rate.

**Table 4:** Effect of ivermectin MDA alone on mosquito density and parity rate in the intervention communities

	*Anopheles* sp.	*p*-value	*Culex* sp.	*p*-value
Density				
Indoor	Intercept	0.170	Intercept	< 0.001
	Exposure to MDA	0.663	Exposure to MDA	0.005*
Outdoor	Intercept	0.249	Intercept	< 0.001
	Exposure to MDA	0.826	Exposure to MDA	0.041*
Parity rate				
Indoor	Intercept	< 0.001	Intercept	< 0.001
	Exposure to MDA	< 0.001*	Exposure to MDA	0.517
Outdoor	Intercept	< 0.001	Intercept	< 0.001
	Exposure to MDA	< 0.001*	Exposure to MDA	0.104

*Statistically significant effect on mosquito density or parity rate

In Table 5, the generalized linear model showed that ivermectin MDA had a significant effect on the density (*p* < 0.001) and parity rate (*p* = 0.003) of indoor *Anopheles* populations in the intervention communities. Rainfall also showed a significant effect (*p* < 0.001) on the density of indoor *Anopheles* populations in the intervention communities whereas the vegetation index showed a significant effect (*p* = 0.031) on the density of the outdoor *Anopheles* population in the intervention communities.

**Table 5:** Generalized linear model showing effects of MDA, rainfall and vegetation index in the intervention communities

	Indoor				Outdoor			
	*B*	SE	CI	*p*-value	*B*	SE	CI	*p*-value
*Anopheles* sp.								
Density								
Intercept	0.134	0.5810	− 1.004 to 1.273	0.319	− 6.375	2.8019	− 11.86 to (− 0.883)	0.029
Exposure to MDA^†^				< 0.001*				0.094
Rainfall	− 0.868	0.2240	− 1.307 to (− 0.429)	< 0.001*	0.838	0.4699	− 0.084 to 1.759	0.075
Vegetation index	2.971	2.1644	− 1.271 to 7.213	0.170	24.235	11.213	2.258 to 46.211	0.031*
Parity rate								
Intercept	5.011	0.3920	4.242 to 5.779	< 0.001	5.985	2.0793	1.910 to 10.060	0.048
Exposure to MDA^†^				0.003*				0.457
Rainfall	− 0.127	0.1510	− 0.423 to 0.169	0.399	− 0.333	0.3487	− 1.017 to 0.350	0.339
Vegetation index	− 1.732	1.4594	− 4.593 to 1.128	0.235	− 5.799	8.3210	− 22.108 to 10.510	0.486
*Culex* sp.								
Density								
Intercept	− 1.801	0.5176	− 2.816 to (− 0.787)	0.216	0.487	1.1259	− 1.720 to 2.694	0.996
Exposure to MDA^†^				0.099				0.650
Rainfall	− 0.025	0.2189	− 0.454 to 0.404	0.910	0.302	0.4486	− 1.181 to 0.577	0.501
Vegetation index	− 0.817	1.7724	− 4.290 to 2.657	0.645	3.857	4.3361	− 4.641 to 12.356	0.374
Parity rate								
Intercept	4.313	0.1723	3.976 to 4.651	< 0.001	4.406	0.5203	3.387 to 5.426	< 0.001
Exposure to MDA^†^				0.434				0.442
Rainfall	0.034	0.0729	− 0.109 to 0.176	0.644	− 0.093	0.1990	− 0.483 to 0.297	0.639
Vegetation index	1.115	0.5900	− 0.042 to 2.271	0.059	− 1.045	1.9234	− 4.814 to 2.725	0.587

*SE* standard error, *CI* 95% confidence interval

*Statistically significant effect on mosquito density or parity rate

^†^Categorical variable

## Discussion

Before ivermectin MDA, the densities of the indoor and outdoor populations of *Anopheles* mosquitoes were more than five times lower in the intervention communities than in the control communities. This difference between the indoor populations was statistically significant. In contrast, there was no statistically significant difference in the densities of *Culex* between the two study areas. A similar observation was reported in a longitudinal study carried out in northeastern Tanzania, where a decline in the density of *Anopheles* was observed while the abundance of *Culex* mosquitoes remained unaffected [15, 16]. Derua et al. [17] attributed this observation in Tanzania to the use of ivermectin for the control of onchocerciasis and LF in the area for > 10 years. In our study, the intervention communities had received ivermectin MDA for at least 5 years while there was no MDA in the control communities within the same period. Although we did not conduct a longitudinal investigation, the baseline *Anopheles* density differences observed between the intervention and control communities could be hypothetically attributed to long-term ivermectin mass drug administration for onchocerciasis and LF control.

A reduction in the daily survival rate is recognised as a primary effect of ivermectin MDA on malaria vectors in insectary-based studies [18]. In our field-based evaluation, this translated to a reduction in the abundance of malaria vectors after MDA. In the intervention communities, the indoor density of *Anopheles* mosquitoes reduced 2–3 days after ivermectin MDA, and a rebound became noticeable after 2 weeks. These changes in the malaria vector abundance followed a consistent pattern in the two intervention communities. In contrast, the abundance of *Culex* sp. increased significantly in the intervention communities 2–3 days after ivermectin MDA. Studies have shown that unlike *Anopheles* sp., the other mosquito vectors are not readily susceptible to the concentrations of ivermectin found in human blood after MDA with the currently recommended safe dosage of the drug [17, 19]. This indicates that factors other than the MDA likely account for the observed reduction in the indoor density of *Culex* sp. in one of the intervention communities.

Interestingly, we observed a reduction in the density of *Anopheles* mosquitoes in the control communities at a magnitude similar to the reduction observed in the intervention communities after MDA. This investigation was carried out in a study area where the spatial scale may make it possible for both humans and mosquitoes to move between, at least, one of the intervention communities and its pair-matched control community [20]. In addition, the proximity of the control communities to the surrounding villages, where the annual ivermectin MDA programme proceeded normally during the study may contribute to the observed reduction in the density of *Anopheles* in the control communities. However, it is also notable that the study area is located within the forest ecozone of Nigeria, where the contamination of vector populations between the control communities and the other surrounding villages may be limited by natural environmental barriers. In addition, the absence of a significant change in the parity rate of *Anopheles* mosquitoes in the control communities 2–3 days after ivermectin MDA contradicts the possible effect of contamination from the surrounding villages where MDA was conducted. The factors responsible for this observation in the control communities are not clear at the moment, which calls for further investigation.

The short-lived reduction in the indoor density of *Anopheles* sp. in the two intervention communities is similar to observations from previous studies on the life span or survival rate of *Anopheles* mosquitoes captured from villages treated with ivermectin. In a study carried out in Senegal, Liberia and Burkina Faso, reduction in the daily survival rate of *An. gambiae* was only observable within the first week after MDA [18]. Also, a clinical trial in Burkina Faso showed that mortality increased for up to 7 days in *An. gambiae*, which were fed with blood from individuals treated with a single dose of ivermectin [5]. It is generally believed that the recommended dose of ivermectin for onchocerciasis and LF control programmes will not have long-lasting lethal effects on malaria vectors [19, 21].

On the other hand, disruption of the age structure of *Anopheles* mosquitoes can last up to 3 weeks after ivermectin MDA [18, 20]. In our study, the proportion of parous (older) female *Anopheles* of the indoor population also remained significantly reduced by > 20% in the intervention communities 2 weeks after MDA. This significant shift to a younger population of female *Anopheles* sp. has important implications for malaria transmission because the older or parous female mosquitoes are commonly the infectious vectors [19]. It has been indicated that the impact on the mosquito population age structure may be the main mechanism by which ivermectin MDA affects malaria transmission [4]. An ivermectin-treated blood meal kills most of the infectious mosquitoes leaving behind a population predominated by young nulliparous mosquitoes, which require some time to become infectious [19].

Importantly, the reduction in the indoor density of *Anopheles* mosquitoes by 29.07% in the intervention communities 2–3 days after MDA was statistically significant. However, our models suggest that ivermectin MDA did not show a clear effect on the indoor density of the *Anopheles* mosquitoes. Using the relative exposure to ivermectin during the three sampling intervals, the model indicated that ivermectin exposure alone showed no significant effect on the indoor density of *Anopheles* sp. but demonstrates a significant effect when other factors are considered. On the other hand, ivermectin exposure showed a clear effect on the parity rate of the indoor *Anopheles* mosquitoes in the intervention communities in our generalized linear models. Hence, although ivermectin MDA may not have a long-lasting impact on the density of *Anopheles* sp., it reduced the proportion of parous older mosquitoes, which are critical for the transmission of malaria in the localities.

Our findings suggest that ivermectin MDA will have more profound effects on the density and parity rate of the *Anopheles* populations at higher MDA coverage. In Amini, where the coverage of ivermectin MDA was significantly higher, the density and parity rate of the indoor *Anopheles* population reduced by 63% and 57% respectively 2–3 days after MDA. These reductions in the density and parity rate were higher and statistically significant compared to Kugba-Ajagbe where the MDA coverage was lower. Therefore, higher MDA coverage in the intervention communities has the potential to enhance the mosquitocidal effects of ivermectin compared to the current observations. Interestingly, there was a prolonged reduction in the indoor density and parity rate of *Anopheles* mosquitoes in Kugba-Ajagbe, where a higher proportion of female individuals was treated with ivermectin. This finding is corroborated by the trial conducted in Burkina Faso, where the lethal effects of ivermectin were stronger and more prolonged in the *Anopheles* mosquitoes that fed on female individuals compared to the males because the female individuals have higher adipose tissue mass, which aids the accumulation of ivermectin and also acts as a slow-release mechanism for the drug [5].

Ivermectin did not show an effect on the outdoor populations of malaria vectors in our study. Unlike the indoor *Anopheles* populations, the outdoor density of *Anopheles* mosquitoes in the intervention communities increased 2–3 days after ivermectin MDA. Although the parity rate of the outdoor *Anopheles* population decreased progressively after MDA, our models showed no significant effects of ivermectin MDA on either the density or parity rate of outdoor *Anopheles* populations. In addition, variations in the density and parity rate of the outdoor *Anopheles* populations in the two intervention communities did not show corresponding patterns as observed for the indoor populations. In this study, samples of the outdoor mosquito populations were collected using the CDC light trap method. Several limitations of this method for outdoor sampling of malaria vectors have been highlighted in different settings, including the possibility of underestimating the density of host-seeking mosquitoes, which might have affected the number of outdoor mosquito samples collected during this study [22]. Although our CDC light traps were augmented with improvised CO_2_ baits to improve performance, the baits may have operated for a limited period during mosquito collection. Therefore, our findings may provide limited information on the impact of ivermectin MDA on the outdoor populations of malaria vectors in the communities where the study was carried out.

In Nigeria, sibling species of the *An. gambiae* complex such as *An. gambiae* *s.s.* and *An. arabiensis* are recognised as the principal vectors of malaria, and emerging shifts toward zoophily, outdoor biting and exophily have been reported, indicating an increasing challenge of residual transmission of malaria in the country [23, 24]. Although the morphological identification carried out in our study does not include information on the species of *Anopheles* mosquitoes collected, previous investigations carried out in locations close to our study area showed that the *Anopheles* samples collected comprised only the known vectors of malaria in Nigeria [25, 26]. Our findings support the need for further investigation of ivermectin MDA as a complementary tool for malaria control. Ivermectin is distributed annually or semi-annually through the mass drug administration programmes for the control of onchocerciasis and lymphatic filariasis. This provides an opportunity to synergize the existing NTD programmes with an ivermectin MDA programme targeted at malaria elimination. The Ivermectin Roadmap outlines the processes needed for the deployment of ivermectin as a vector control tool by 2024, including the need to establish a target population and the level of community uptake required [27]. Our study has provided evidence highlighting the importance of ivermectin therapeutic coverage in achieving an effective reduction in *Anopheles* mosquitoes as well as the transmission of malaria.

## Conclusions

The abundance of *Anopheles* mosquitoes was significantly lower in the intervention communities where the annual ivermectin MDA round has been ongoing for a long period. The density of malaria vectors was reduced in the two intervention communities after ivermectin MDA for onchocerciasis and LF. Although the density of *Anopheles* mosquitoes may rebound quickly after single-dose ivermectin MDA, disruption of the age structure and its implication for malaria transmission will likely last for a longer time. Overall, a high MDA coverage targeting a high proportion of female inhabitants in a community will maximize the benefits of ivermectin as a control tool for malaria vectors.

## Data Availability

The datasets for climatic and environmental variables generated and analysed during the current study are publicly available in the remote sensing data repositories cited. The primary datasets generated and analysed are available from the corresponding authors on reasonable request.

## Abbreviations

MDA: Mass drug administration
LF: Lymphatic filariasis
LLIN: Long-lasting insecticidal net
IRS: Indoor residual spraying
LGA: Local government area
WHO: World Health Organization
DDT: Dichlorodiphenyltrichloroethane
CDD: Community-directed distributor
